# Development of an Analytical Model for Predicting the Shear Viscosity of Polypropylene Compounds

**DOI:** 10.3390/polym17020126

**Published:** 2025-01-07

**Authors:** Lukas Seifert, Lisa Leuchtenberger-Engel, Christian Hopmann

**Affiliations:** Institute for Plastics Processing (IKV) in Industry and Craft, RWTH Aachen University, Seffenter Weg 201, 52074 Aachen, Germany

**Keywords:** polypropylene, shear viscosity, predictive modelling, additives, chalk, impact modifiers, peroxides, analytical model, polymer blends

## Abstract

The need for an efficient adaptation of existing polypropylene (PP) formulations or the creation of new formulations has become increasingly important in various industries. Variations in viscosity resulting from changes in raw materials, fillers, and additives can have a significant impact on the processing and quality of PP products. This study presents the development of an analytical model designed to predict the shear viscosity of complex PP blends. By integrating established mixing rules with novel fitting parameters, the model provides a systematic and efficient method for managing variability in PP formulations. Experimental data from binary and multi-component blends were used to validate the model, demonstrating high prediction accuracy over a range of shear rates. The proposed model serves as a valuable tool for compounders and manufacturers to optimise PP formulations and develop new recipes with consistent processing and product quality. Future work will include industrial-scale trials and further evaluation against advanced machine learning approaches.

## 1. Introduction

Plastics have become an elementary component of modern society, valued for their adaptability, cost-effectiveness, and durability. In Europe, the packaging sector is the largest consumer of plastics, accounting for around 40% of total demand, followed by the construction sector with around 20% and the automotive industry, which uses around 9-10% of all plastics. Among these materials, polypropylene (PP) stands out as the most widely used polymer, particularly in packaging, due to its balance of mechanical properties and ease of processing [1,2]. Its versatility extends beyond packaging to applications in construction materials and automotive components, making it a key material for a range of industries. Given its extensive use, ensuring consistent material properties across applications is critical for efficient processing.

A key factor in determining the processability of PP and other polymers is shear viscosity, which measures the resistance of the material to flow under shear deformation. Shear viscosity is influenced by a number of factors including molecular weight, temperature, additives, and the shear rate itself [3,4]. Typically, shear viscosity is characterised using a capillary rheometer, which measures flow pressure differences at different shear rates. Typical shear rates in polymer processes such as extrusion range from 1 s^−1^ to 1000 s^−1^, while injection moulding involves much higher shear rates, between 100 s^−1^ and 100,000 s^−1^ [3]. The interplay between viscosity and shear rate is critical for efficient and high-quality polymer processing, including extrusion, injection moulding, and blow moulding.

Variations in shear viscosity pose challenges in numerous manufacturing processes [5,6,7,8]. In injection moulding, for example, if the viscosity is too low, the mould can be overfilled, while if it is too high, the mould can be incompletely filled—both of which require costly adjustments to moulds and processing conditions [5,6]. Similarly, in film extrusion, consistent viscosity is essential to avoid defects and maintain smooth production [7]. These challenges are even more pronounced when processing recyclates, which tend to have variable properties due to inconsistencies in source materials, previous use, and contamination levels [9,10,11,12,13].

However, recyclates are not the sole source of variability. Even when using virgin polymers, factors such as batch-to-batch variations, changes in suppliers, or the need to switch to different polymer grades for economic or supply chain reasons can lead to changes in viscosity. Additionally, adjustments in the formulation, such as the inclusion of different fillers and additives from other suppliers to modify the polymer’s mechanical properties or to achieve specific performance criteria, can further influence the compound’s viscosity.

There are two main approaches for solving the problems associated with different viscosities. One approach focuses on adjusting the processing parameters of the various manufacturing machines. In this area, extensive research is being carried out in the development of assistance systems and methods designed to systematically adjust processing conditions to compensate for viscosity variations. In injection moulding, for example, advanced process control strategies for the injection and holding pressure phases have been developed to maintain consistent processing points even when viscosities vary [14,15]. In blown film extrusion, researchers are investigating ways to compensate for viscosity changes within the melt pre-distributor [16]. In addition, commercial solutions from machine manufacturers offer automated adjustments to processing parameters, enabling smoother production despite variations in input material viscosity [17].

While such assistance systems can effectively manage production processes in the presence of varying viscosities, they do not address other quality parameters of the material that may be critical to the final product. For example, processing a recyclate batch with a different composition that results in a lower tensile modulus or impact strength may be optimised in terms of flow characteristics during processing, but the mechanical properties of the product could still be compromised.

To guarantee the processability of compounds and ensure the requisite mechanical properties for the final product, it is necessary to modify the recipe or compound formulation in such a way that all essential properties of the compound are maintained, irrespective of variations in the quality of the input stream. This approach requires the development of reliable models capable of predicting these properties in relation to the base materials, fillers, and additives used [10,11,12,13,18,19]. With the use of such models, it becomes possible to proactively adjust formulations to ensure consistency in both processing and product quality, even in the face of fluctuating input materials.

## 2. Development and Modification of Compound Recipes

Traditionally, the development and tuning of PP formulations has relied on empirical methods and the expertise of experienced compounders. While valuable, this procedure is inherently iterative and time-consuming, and often lacks the precision required to handle the complex interactions within PP blends, especially those containing recycled materials or a multitude of additives [10,11,20,21,22].

Without a systematic approach, compounders face challenges in achieving consistent quality in their formulations, as varying input streams can lead to unpredictable changes in viscosity. Formulation adjustments typically require extensive trial and error, which is not only resource-intensive, but it also limits the ability to quickly adapt to new or fluctuating material streams [21,22].

In recent years, data-driven approaches, including machine learning (ML) and artificial intelligence (AI), have shown promise in predicting and optimising the properties of polymer blends. Methods such as artificial neural networks (ANNs) have been used to predict a multitude of compound properties [23,24]. For example, Lopez-Garcia et al. demonstrated the use of ANNs to predict the mechanical properties of fibre-reinforced compounds containing recycled fibres, achieving R^2^ values of up to 0.96 [23]. Other studies have used ANNs to optimise compound formulations for specific properties, such as colour and impact strength in polyamides [24].

Despite their success, these AI and ML approaches have their limitations. They require large, comprehensive datasets that include detailed information on formulation components, processing conditions and thoroughly characterised material properties [25,26]. Building such datasets often requires extensive and costly experimentation, making it difficult for many companies to implement these methods [22,25,26]. Furthermore, while AI models can offer high predictive accuracy, they often lack interpretability, making it difficult to understand the underlying relationships between input parameters and predicted properties.

### Proposed Aim of This Paper

Given the limitations of traditional empirical methods and AI models, there is a need for a more adaptable and interpretable approach to model the interactions of the individual components in PP blends. This paper proposes an analytical model specifically designed to predict the shear viscosity of industry orientated PP formulations. By combining established blending rules with novel fitting parameters, this approach aims to provide a systematic and efficient method for predicting the shear viscosity of blends in dependence of their compositions.

Through building on previous success in predicting the tensile modulus of PP blends with high accuracy utilising the same approach, the model developed withing this study requires only a few modifiable fitting parameters and is easily adaptable [10]. This allows rapid adaptation to new formulation components, making it highly practical for industrial use, where consistent product quality is critical.

## 3. Experimental

To obtain experimental data to develop the model to predict the shear viscosity as a function of the composition of blends, additives, and fillers, a variety of compound formulations were identified and produced.

### 3.1. Materials and Characterisation

The experiments utilised two virgin homopolymer PP grades, supplied by Lyondell Basell (Rotterdam, The Netherlands) and the Saudi Basic Industries Corporation (SABIC, Riyadh, Saudi Arabia). Both polymers differ significantly, with 505P having an overall lower viscosity than HP548R. The melt flow rate (MFR) values, an indicator of the polymer’s flow characteristics, were measured according to ISO 1133 at a temperature of 230 °C and a weight of 2.16 kg, typical for PP [27]. The base polymer properties are summarised in Table 1.

The selected additives and fillers represent a range of components commonly employed in different PP formulations. To specifically adjust the viscosity of PP, the peroxide masterbatch CR5P from Polyvel Europe GmbH (Jork, Germany) was used [18]. Peroxide additives are commonly used to modify the molecular structure of polymers, thereby affecting their rheological properties [28]. Additionally, the impact modifier masterbatch Engage 8200 supplied by DOW Inc. (Midland, MI, USA) was included to enhance the impact strength of the PP compounds, an aspect not directly linked to viscosity but potentially influencing it [29].

Fillers such as chalk are frequently added to PP compounds to improve their tensile modulus or reduce cost. For this study, two types of chalk differing in their grain size provided by OMYA GmbH (Oftringen, Switzerland) were used: Omyalite 95 T (fine) and Omyalite 50 H (coarse).

**Table 1:** Base polymers used in the investigation [30,31].

Designation	Material Name	MFR [g/10 min]
vH2	505P	2.0
vH23	HP548R	23.0

### 3.2. Laboratory Equipment for Compounding

Compounding was carried out on a co-rotating twin screw extruder (Coperion GmbH, Stuttgart, Germany) with a screw diameter of 26 mm. Four different base compound compositions were used in all trials, as detailed in Table 2. The machine temperature was maintained at 210 °C. The screw elements consisted of conveying elements with a combination of kneading and mixing elements at the beginning of the process to plasticise the polymers. The compounding process was carried out at a constant screw speed of 300 min^−1^ and a targeted throughput of 15 kg/h.

The different percentages of fillers and additives are shown in Table 3. Each additive or filler was used individually in compounding with the four base compound compositions, with no compound containing more than one filler or additive. The boundaries for the used percentages were chosen based on recommendations of the material suppliers.

The shear viscosity of the compounds was measured at a temperature of 230 °C using three round capillaries (diameter 1 mm) with lengths of 20 mm, 10 mm, and 5 mm on a high-pressure capillary rheometer (RHEOGRAPH 50, GÖTTFERT Werkstoff-Prüfmaschinen GmbH, Buchen, Germany). The use of three capillaries allowed the Bagley correction to be applied to compensate for inlet and outlet pressure losses, ensuring accurate viscosity measurements for each compound even though each compound was measured only once [32].

## 4. Development of Partial Models for the Prediction of the Shear Viscosity Curve

This section outlines the development of several partial models aimed at predicting the viscosity curves of different compounds as a function of their formulation. The modelling begins with the development of a model for binary polymer blends and extends to models that consider the effects of additives such as chalk, impact modifier, and peroxide.

### 4.1. Development of a Model to Predict the Shear Viscosity of Binary Homo Polymer Blends

The shear viscosity of the various blends was measured at fixed shear rates of 51 s^−1^, 102 s^−1^, 204 s^−1^, 408 s^−1^, 816 s^−1^, and 1630 s^−1^. However, to investigate viscosity at specific processing shear rates, interpolation between measurements may be required. A variety of models can be used to describe the shear viscosity curve. In a previous investigation, several models for predicting the shear viscosity of binary and ternary blends of PP were investigated in detail [11]. The Carreau model with η being the shear viscosity in dependence of the shear rate $\dot{\gamma }$, given by Equation (1), was found to be best suited for describing the viscosity curve for the fixed shear rates used in the measurements.

(1) $$\eta \left(\dot{\gamma }\right)=\frac{A}{{\left(1+B\dot{\gamma }\right)}^{C}}$$

**Figure 1:**
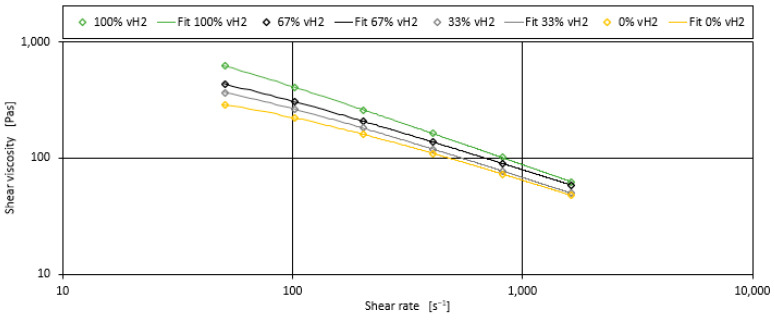
Measurements and shear viscosity curves derived with the Carreau model for the four binary blends without any additives or fillers.

For each blend, the three parameters of the Carreau model, A, B, and C, were fitted to the measured data to minimise the squared error between prediction and measurement. Figure 1 shows the measured data alongside the curve calculated using the Carreau model for the four binary blends without additives and fillers. 

Various mixing rules can describe the shear viscosity of a binary blend at a defined shear rate [11]. The linear blending rule, identified in previous studies as effective for PP, is given by Equation (2) [11].

(2) $$\eta_{mix,\;blend}\left(\dot{\gamma }\right)=x_{1}\eta_{1}\left(\dot{\gamma }\right)+x_{2}\eta_{2}\left(\dot{\gamma }\right)$$

**Figure 2:**
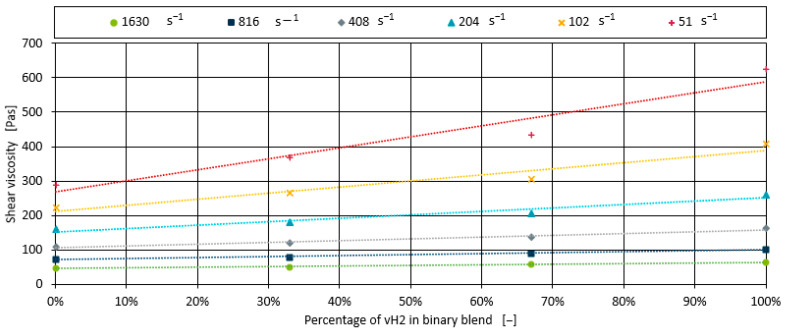
Measured shear viscosity for fixed shear rates plotted with a linear mixture model (dotted lines).

Here, $x_{1}$ and $x_{2}$ are the percentual shares of both polymers in the binary blend with $\eta_{1}\left(\dot{\gamma }\right)$ and $\eta_{2}\left(\dot{\gamma }\right)$ being their individual shear viscosity for the given shear rate $\dot{\gamma }$. Figure 2 shows the measured shear viscosities for different shear rates alongside the linear model (Equation (2)). To minimise prediction error, the linear model was fitted to the data, resulting in some deviation from the measured data. The model shows high accuracy for shear rates from 204 s^−1^ to 1630 s^−1^, effectively fitting all measurement points. However, at lower shear rates of 51 s^−1^ and 102 s^−1^, the shear viscosity measurements, e.g., for the 66% vH2 blend, decrease slightly.

The R^2^ value (coefficient of determination) and the Mean Percentage Error (MPE) are used to assess the predictive accuracy of the models for the binary mixtures as well as for the models developed in the following subchapters.

The R^2^ statistic measures the proportion of variance in the dependent variable that is explained by one or more independent variables in a regression model. An R^2^ value of 1 indicates a perfect fit, i.e., the predictions of the model exactly match the observed data. Conversely, an R^2^ of 0 indicates that the model does not explain any of the variability in the dependent variable. As the shear viscosity values cover a range of magnitudes, calculating an overall R^2^ series may lead to biassed conclusions, as the prediction accuracy at lower shear rates, such as 51 s^−1^ or 102 s^−1^, is more heavily weighted by the higher viscosities. Therefore, R^2^ values are calculated individually for each measured shear rate. However, the MPE can be used to compare the prediction accuracies for the different shear rates.

Table 4 summarises these metrics for the linear mixing model over all blends. The R^2^ values are close to 1.0, with a minimum of 0.9978 for the shear rate of 102 s^−1^, while the MPE remains below 1%, indicating high prediction accuracy. These results are congruent with other studies confirming the linear mixing rule to be suitable for predicting the shear viscosities of PP blends [11,33,34].

### 4.2. Development of a Model to Predict the Shear Viscosity of Binary Homo Polymer Blends with Peroxide Masterbatch

**Figure 3:**
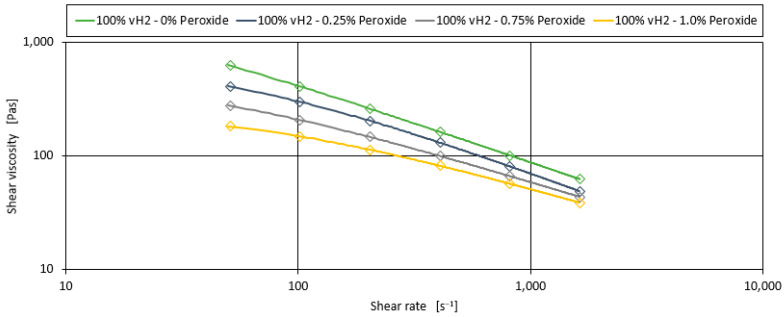
Shear viscosity curves for the blend of 100% vH2 with the addition of various concentrations of peroxide additive.

This section examines the effect of the addition of a peroxide containing masterbatch on the tensile modulus of the blends. Figure 3 shows the fitted Carreau curves for 100% vH2 with varying concentrations of peroxide additive (0.25%, 0.75%, and 1.00%). The data show that the shear viscosity decreases with increasing peroxide content at all shear rates, as expected due to the shortening of the PP chain length [27].

**Figure 4:**
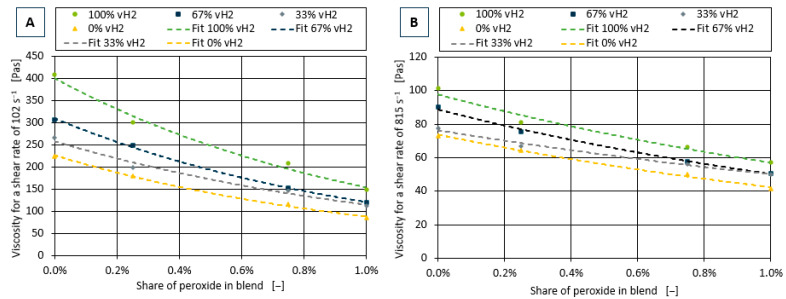
Shear viscosity curves for all blends in dependence of the share of peroxide additive in the blend for the two exemplary shear rates of 102 s^−1^ (**A**) and 815 s^−1^ (**B**).

To determine a model capable of describing the influence of the peroxide additive, the viscosity in dependence of the peroxide additive content was investigated for each of the measured shear rates. This can be seen for the exemplary shear rates of 102 s^−1^ and 815 s^−1^ in Figure 4.

**Figure 5:**
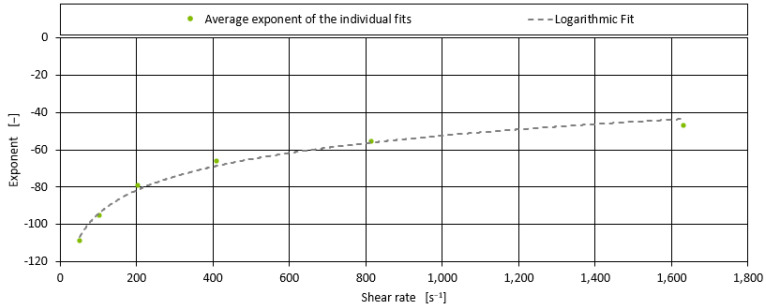
Correlation of the average exponents of the exponential fit performed for the various shear viscosities for the peroxide containing mixtures in dependence of the shear rate.

After examining the measured data for each of the shear rates, it was found that an exponential function fits the measured data very well. This exponential function depends on the shear viscosity of the blend without peroxide and the amount of peroxide multiplied by a constant in the exponent of the exponential function. By examining the exponential functions for all blends, it was found that the exponent of the individual fits was very similar for all four blends for each shear rate. In order to develop an expression for the exponential function that is universally applicable to all shear rates, a function must be found to describe this exponent of the exponential functions as a function of the shear rate. The correlation for this exponent of the exponential function for all shear rates is shown in Figure 5.

It can be seen that all values for the exponents fit quite well with a logarithmic trend. With this correlation, a universal formula can be derived to predict the shear viscosity for all blends as a function of the shear rate and the viscosity for a given shear rate for the blend without peroxide, according to Equation (3).

(3) $$\eta_{mix,\;peroxide}\left(\dot{\gamma ,}x_{Peroxide}\right)=\eta_{mix,\;blend}\left(\dot{\gamma }\right)*e^{x_{Peroxide}*(p_{1}*\ln\left(\dot{\gamma }\right)+p_{2})}$$

The coefficients $p_{1}$ and $p_{2}$ are the only parameters required to fit the model for all data containing peroxide as an additive. They were fitted to minimise the overall MPE and were determined to be $p_{1}$ = 19.485 and $p_{2}$ = −187.147. The individual R^2^ and MPE for all measured shear rates are shown in Table 5.

In general, the accuracy of the model is lower than that of the binary blend with no additives or fillers. The best R^2^ value was obtained for 408 s^−1^ with a value of 0.9798, while the worst value was obtained for 1630 s^−1^ with a value of 0.9096. Similar results can be seen for the MPE, with a minimum of 3.244% and a maximum of 9.538%. One reason for this may be inaccuracies in the dosing equipment during the trials. With a mass throughput of 15 kg/h during the tests, a throughput of 37 g/h had to be set for the test points with 0.25% peroxide additive content. Small deviations in the throughput result in a difference between the assumed and the actual peroxide concentration, which may explain the rather high inaccuracies for the prediction model.

### 4.3. Development of a Model to Predict the Shear Viscosity of Binary Polymer Blends with Chalk

**Figure 6:**
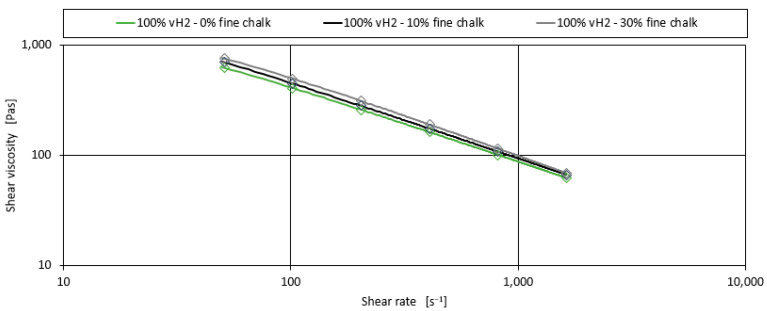
Shear viscosity curves for the blend of 100% vH2 with the addition of fine chalk.

Following the development of the model for binary homopolymer blends with peroxide, this section examines the effect of adding chalk as a filler on the shear viscosity. For the 100% vH2 blend with 10% and 30% fine chalk as filler, the effect of chalk on shear viscosity is shown in Figure 6.

**Figure 7:**
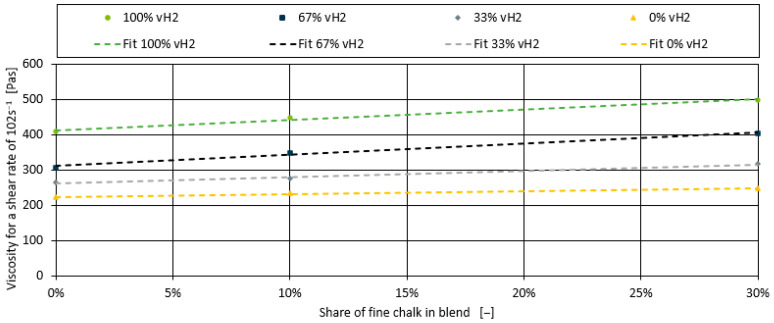
Shear viscosity for shear rate of 102 s^−1^ for the blends containing fine chalk.

Compared to the addition of a peroxide additive, the addition of up to 30% chalk as filler only results in a minor increase in shear viscosity of up to 22%. This can be observed for all blends and for both types of chalk used in the investigation. Nevertheless, the addition of chalk has an influence and will be modelled. Figure 7 illustrates the effect of the fine chalk for the exemplary shear rate of 102 s^−1^.

**Figure 8:**
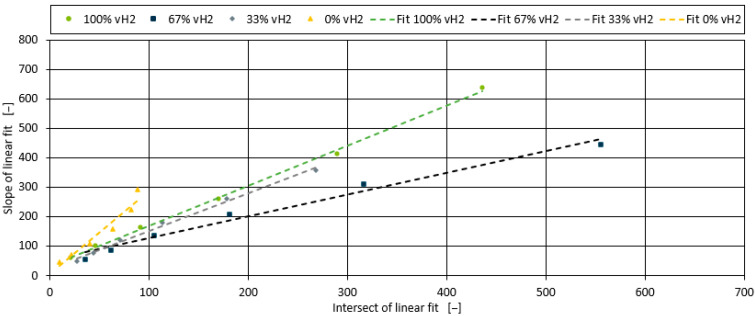
Correlation of slope and intercept of linear fit for all blends and shear rates containing fine chalk.

It can be seen that the effect is linear for all four blends. Similarly to the development of the model for the peroxide additive, the correlation between the slope for the four blends with chalk and the viscosity at 0% chalk (intercept of the four linear fits in Figure 7) is shown in Figure 8. The relationship is again linear. While the slopes of 100% vH2 and 33% vH2 are almost identical, the linear fits for 0% vH2 and 67% vH2 are slightly different. However, compared to the peroxide additive, the chalk has less influence despite its higher filler content. Therefore, a pragmatic modelling approach is taken, and the linear correlation fits in Figure 8 are averaged and reduced to one linear function. This results in inaccuracies in the prediction for the 0% vH2 and 67% vH2 blends, but the trends are still captured.

Through the above approach, the effect of chalk on the shear viscosity curve as a function of compound, shear rate, and chalk content can be described by Equation (4).

(4) $$\eta_{mix,\;chalk}\left(\dot{\gamma ,}x_{Chalk}\right)=\eta_{mix,\;blend}\left(\dot{\gamma }\right)+x_{Chalk}*(c_{1}*\eta_{mix,\;blend}\left(\dot{\gamma }\right)-c_{2})$$

For the mixtures containing the fine chalk, parameters $c_{1\;}$ and $c_{2}$ were fitted in the same way as the parameters of the previous model for the peroxide in order to minimise the MPE. The same procedure was used for the coarse chalk to determine parameters $c_{3}$ and $c_{4}$. The fitted parameters are shown in Table 6.

The calculation of the R^2^ and MPE values for each shear rate for both types of chalk gives the values shown in Table 7. Apart from the R^2^ value of 0.9147 for the fine chalk at the shear rate of 1630 s^−1^, a minimum R^2^ value of 0.9553 is obtained. It can also be seen that the R^2^ values for the coarse chalk are slightly better at all other shear rates. Although the R^2^ values for the shear rate of 1630 s^−1^ appear to be the worst, the MPEs of 2.464% for fine chalk and 1.889% are among the lowest errors, indicating good model prediction accuracy, especially at higher shear rates.

### 4.4. Development of a Model to Predict the Shear Viscosity of Binary Homo Polymer Blends with Impact Modifier

**Figure 9:**
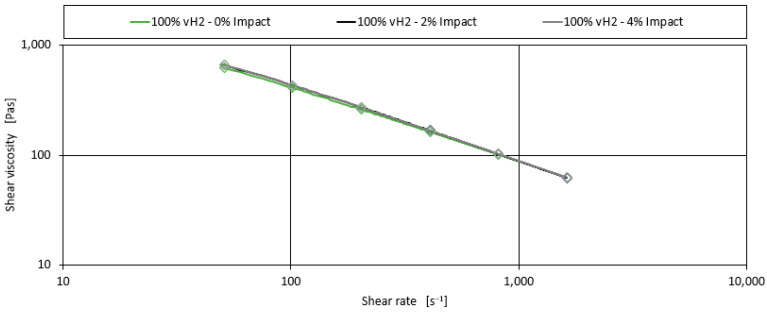
Shear viscosity curves for the blend of 100% vH2 with the addition of impact modifier additive.

Finally, the effect of the impact modifier on the shear viscosity of the blends is investigated. Figure 9 shows the effect of 2% and 4% impact modifier on the shear viscosity curve for 100% vH2. There is almost no effect on the shape of the curve. No significant effect was observed for the other three blends. Therefore, the addition of an impact modifier is neglected, and no separate model is developed.

## 5. Aggregation of Partial Models to an Extensive Model

As shown in the previous sections, individual models were developed to predict the shear viscosity of binary PP blends and the effects of various fillers and additives such as chalk, impact modifiers, and peroxide additives. The next logical step is to integrate these partial models into a single comprehensive model capable of predicting the shear viscosity of more complex blends.

The aggregated model is constructed by combining each partial model through additive contributions to the base model describing the binary blends. This aggregation is represented mathematically by Equation (5):

(5) $$\eta_{mix}(\dot{\gamma })=\eta_{mix,\;blend}+∆\eta_{Peroxide}+∆\eta_{Chalk\;fine}+∆\eta_{Chalk\;coarse}$$

Equations (3) and (4) from the earlier sections are modified to form Equations (6)–(8), describing each additive’s effect in the final model.

(6) $$∆\eta_{Peroxide}=\eta_{mix,\;blend}\left(\dot{\gamma }\right)*e^{x_{Peroxide}*(p_{1}*\ln\left(\dot{\gamma }\right)+p_{2})}-\eta_{mix,\;blend}\left(\dot{\gamma }\right)$$

(7) $$∆\eta_{Chalk\;fine}=x_{Chalk\;fine}*(c_{1}*\eta_{mix,\;blend}\left(\dot{\gamma }\right)-c_{2})$$

(8) $$∆\eta_{Chalk\;coarse}=x_{Chalk\;coarse}*(c_{3}*\eta_{mix,\;blend}\left(\dot{\gamma }\right)-c_{4})$$

These equations include the parameters fitted in the previous sections, where $x_{Chalk\_fine}$, $x_{Chalk\_coarse}$, $x_{Impact}$, and $x_{Peroxide}$ represent the proportions of the respective additives in the mixture. Coefficients $c_{1}$ to $c_{2}$ correspond to the intercept and slope parameters identified for each chalk type, while $p_{1}$ and $p_{2}$ are the fitting parameters for the peroxide model.

After developing the full model by combining these partial models, all parameters were optimised using the full dataset to minimise the overall MPE. The shear viscosity curves used to calculate the viscosity of the blend, $\eta_{1}\left(\dot{\gamma }\right)$ and $\eta_{2}\left(\dot{\gamma }\right)$ were measured. However, in order to ensure the best possible accuracy for the aggregated model, the Carreau parameters according to Equation (1) were also refined during the optimisation process. The final optimised parameters for the full model are shown in Table 8.

**Figure 10:**
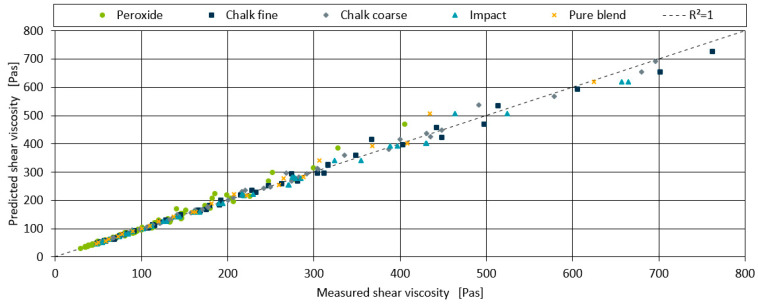
Plot of model prediction and measurements for each data point. The dotted line marks a perfect match between prediction and measurement. The individual additives and fillers are assigned to different colours.

Figure 10 shows the individual predictions of each data point plotted against the measurements. The dotted line in the graph indicates an exact match between prediction and measurement. It can be seen that over the full range of shear viscosities measured, the predictions and measurements of all data points, regardless of their fillers or additives, are in fairly close alignment. The highest percentage deviations occur in the peroxide containing blends, as expected and explained in Section 4.2.

The R^2^ and MPE of all measurements for each shear rate were calculated to assess the aggregated model’s performance, as shown in Table 9. As expected, the R^2^ for the shear rate of 1630 s^−1^ is the lowest, with a value of 0.9553. Nevertheless, for all other shear rates, a minimum R^2^ of 0.9723 up to a maximum R^2^ of 0.9849 was achieved, indicating very good model quality. The prediction error for the highest shear rate of 1630 s^−1^ is the worst, with an MPE of 6.503%, while the best prediction accuracy is for 102 s^−1^ with an accuracy of 2.424%.

## 6. Discussion

In this study, an analytical model was developed to predict the shear viscosity of PP blends consisting of multiple polymers, fillers, and additives. The model combined established blending rules with additional fitting parameters to provide accurate viscosity predictions for complex PP blends.

The performance of the model was evaluated over a range of shear rates and showed good agreement with the measured data. For the binary blends without additives, the model achieved an R^2^ value of up to 0.9994 at a shear rate of 408 s^−1^ and maintained an R^2^ value above 0.9978 across all measured shear rates. This high level of accuracy indicates that the model effectively captures the shear viscosity behaviour of the base polymers.

When extended to include additives such as peroxide, the model accounted for the observed decrease in viscosity with increasing peroxide concentration. The model achieved an R^2^ value of 0.9798 at 408 s^−1^, and although the R^2^ value dropped to 0.9096 at the highest shear rate of 1630 s^−1^, the MPE remained within acceptable limits, indicating reasonable predictive accuracy for blends containing peroxide. For blends containing chalk, both fine and coarse, the model maintained an R^2^ value above 0.9147, demonstrating its ability to predict the viscosity changes induced by fillers with high contents. The overall predictive accuracy of the model for all blends and shear rates was confirmed by an R^2^ value ranging from 0.9553 to 0.9849, with the MPE reaching a maximum of 6.503% at the highest shear rate of 1630 s^−1^.

## 7. Conclusions

The aggregation of partial models into a comprehensive framework allowed for the prediction of the combined effects of multiple additives on the shear viscosity of PP blends.

Future work will focus on validating the model in an industrial setting, specifically through extended trials on a twin-screw extruder using the formulations investigated in this study, as well as additional formulations containing all additives and fillers simultaneously. These trials will further test the robustness of the model and its ability to handle the complexities of real processing conditions. In addition, a comparative analysis with advanced machine learning approaches is planned to benchmark the performance of the analytical model. This comparison will help to identify any potential advantages or limitations over data-driven models and provide insights into how these different approaches could be combined or used in a complementary manner to optimise polymer processing. Lastly, the model developed within this paper will be applied on other types of polymers and polymer mixtures to determine whether it is generally applicable.

In conclusion, this analytical model represents a significant step toward a more adaptable and systematic approach to the management of shear viscosity in PP blends. Its strong predictive capability, as demonstrated by the high R^2^ values and low MPEs across different formulations and shear rates, underlines its potential utility in developing and adjusting PP formulations with greater efficiency and accuracy.

## Figures and Tables

**Table 2:** Blend composition for the main trials with vH2 (X_1_)–vH23 (X_2_).

**X_1_**	0%	33%	67%	100%
**X_2_**	100%	67%	33%	0%

**Table 3:** Blend, filler, and additive compositions for the blend of vH2–vH23.

Designation	Material	Percentages			
Chalk fine	Omyalite 95 T	10%		30%	
Chalk coarse	Omyalite 50 H	10%		20%	
Impact modifier	Engage 8200	2%		4%	
Peroxide additive	Polyvel CR5P	0.25%	0.75%		1.00%

**Table 4:** R^2^ and MPE for the linear model of the four binary blends without additives or fillers.

Shear Rate	1630 s^−1^	816 s^−1^	408 s^−1^	204 s^−1^	102 s^−1^	51 s^−1^
R^2^	1.0000	1.0000	0.9994	0.9996	0.9978	0.9989
MPE	0.003%	0.002%	0.373%	0.355%	0.978%	0.807%

**Table 5:** R^2^ and MPE for the model predicting the shear viscosity for the different shear rates for the blends containing peroxide.

Shear Rate	1630 s^−1^	816 s^−1^	408 s^−1^	204 s^−1^	102 s^−1^	51 s^−1^
R^2^	0.9096	0.9711	0.9798	0.9793	0.9706	0.9355
MPE	4.564%	3.244%	3.752%	4.450%	6.040%	9.538%

**Table 6:** Fitted parameters for both the blends with fine and coarse chalk.

**Parameter**	$c_{1}$	$c_{2}$	$c_{3}$	$c_{4}$
Value	0.5191	16.3861	0.7197	23.2449

**Table 7:** R^2^ and MPE for the model predicting the shear viscosity for the different shear rates for the blends containing both types of chalk.

Chalk Type	Shear Rate	1630 s^−1^	816 s^−1^	408 s^−1^	204 s^−1^	102 s^−1^	51 s^−1^
Fine	R^2^	0.9147	0.9638	0.9699	0.9656	0.9612	0.9553
	MPE	2.464%	2.091%	2.574%	3.124%	4.059%	5.620%
Coarse	R^2^	0.9578	0.9707	0.9694	0.9679	0.9658	0.9597
	MPE	1.889%	1.559%	2.005%	2.620%	3.383%	5.162%

**Table 8:** Fitted parameters for the aggregated model.

Parameter	Value	Parameter	Value
$A_{1}$	430.699	$p_{1}$	19.478
$B_{1}$	0.00183	$p_{2}$	−185.824
$C_{1}$	0.64460	$c_{1}$	0.606
$A_{2}$	251.964	$c_{2}$	16.791
$B_{2}$	0.01303	$c_{3}$	0.618
$C_{2}$	0.69068	$c_{4}$	15.648

**Table 9:** R^2^ and MPE for the aggregated model predicting the shear viscosity for the different shear rates for all blends.

Shear Rate	1630 s^−1^	816 s^−1^	408 s^−1^	204 s^−1^	102 s^−1^	51 s^−1^
R^2^	0.9553	0.9820	0.9849	0.9838	0.9809	0.9723
MPE	6.503%	4.273%	3.327%	2.811%	2.424%	3.037%

## Data Availability

All data presented in this study are available on request from the corresponding author.
